# Machine learning-based typing of *Clostridium botulinum* group III by FT-IR spectroscopy

**DOI:** 10.1128/spectrum.01562-25

**Published:** 2026-01-15

**Authors:** Ilenia Drigo, Angela Guolo, Alessia Rizzardi, Miriam Cordovana, Manuel Garbuio, Elena Tonon, Marco Vedana, Luca Zandonà, Luca Bano

**Affiliations:** 1 SCT2- Laboratory of Treviso, Istituto Zooprofilattico Sperimentale delle Venezie 83372, Fontane di Villorba, Italy; 2 Bruker Daltonics GmbH and Co, Bremen, Germany; Quest Diagnostics Nichols Institute, Chantilly, Virginia, USA

**Keywords:** Botulinum neurotoxin-producing clostridia, FT-IR spectroscopy, serotyping

## Abstract

This study aimed to investigate the utility of Fourier-Transform Infrared Spectroscopy (FT-IRS) for differentiating *Clostridium botulinum* (*C. botulinum*) based on its botulinum neurotoxin (BoNT)-encoding gene type and its potential as an epidemiological tool for investigating botulism outbreaks. A total of 110 botulinum neurotoxin-producing clostridia (BNPC) strains, including reference, animal isolates, and human outbreak strains, were analyzed in four replicates using the IR Biotyper system (IRBT). Samples preparation was carried out according to the manufacturer’s instructions. Similarity analysis was performed by hierarchical cluster analysis (HCA), principal component analysis (PCA), and linear discriminant analysis (LDA). The artificial intelligence capabilities of the IRBT software were applied to develop a classifier for *C. botulinum* differentiation at toxin-serotype or subtype level. HCA, PCA, and LDA showed a good clustering of strains belonging to the same type. In accordance with the lineages evidenced in whole-genome sequencing (WGS) studies, types A, B, and F BNPC appeared clearly separated from types C, CD, DC, and D. Considering only C, CD, DC, and D types, the highest discriminatory power is achieved in the wavenumber range 1,800–1,500 cm^−1^. Four different clusters were detected. Support vector machine algorithm with linear kernel (Linear SVM) showed the highest accuracy of discrimination at the BoNT type level with an accuracy of 97%. Although these preliminary results need to be confirmed with a higher number of strains, the IRBT system proved to be a very promising, user-friendly, and cost-effective tool for *C. botulinum* typing, and the application of machine learning algorithms represents a novel approach for BNPC typing.

**IMPORTANCE:**

Botulism outbreaks represent a significant threat to public and animal health. Rapid and accurate typing methods are essential for effective epidemiological investigations, source tracing, and the implementation of appropriate control measures. Current methods for botulinum neurotoxin serotyping are often time-consuming, expensive, and require specialized expertise. Our research demonstrated that FT-IRS, a rapid, user-friendly, and cost-effective technique already well established in microbiology for broader bacterial characterization, can be successfully adapted for this crucial task. The use of a commercially available system like the IRBT significantly enhances the potential for widespread adoption of this methodology in routine diagnostics and surveillance.

## INTRODUCTION

Botulism is a potentially fatal neurological disease affecting both humans and animals caused by the action of botulinum neurotoxin (BoNT), renowned as the most potent biological substance known (1). BoNTs are produced by gram-positive, anaerobic endospore-forming bacteria, belonging to *Clostridium* genera (*Clostridium botulinum*, *Clostridium butyricum*, *Clostridium baratii*, *Clostridium argentinense*, and *Clostridium sporogenes*) (2). In recent years, BoNT-like encoding genes have also been detected in the genome of non-*Clostridium* species, such as *Weissella oryzae* (3), *Enterococcus* spp. (4), and *Chryseobacterium piperi* (5). Eight BoNT toxin types have been described, from A to G, and the recently discovered BoNT/X (1, 6). Additionally, mosaic or chimeric toxins are also known, such as BoNT/FA, initially called BoNT/H as thought to be a new serotype, and BoNT/CD and DC (7, 8). BoNT/A, B, E, and F mainly cause human disease, whereas BoNT/C and D and their mosaic forms BoNT/CD and BoNT/DC have been shown to affect mostly animals (3, 9, 10). BoNT/CD possesses a C-terminal heavy chain similar to BoNT/C toxin, but the light chain and the N-terminal heavy chain are almost identical to BoNT/D. Conversely, BoNT/DC consists of the light chain of BoNT/C and the heavy chain of BoNT/D (8, 11).

FT-IRS is a phenotypic method commonly employed in chemistry to determine the molecular composition of a wide range of sample types. The operational principle of FT-IRS relies on the exploitation of distinct absorption characteristics exhibited by covalent bonds within different functional groups in the infrared region. This rapid, simple, inexpensive, and high-throughput technique has recently been successfully applied in various microbiology fields, yielding significant results and revolutionizing aspects of microbiological research and diagnostics (12). The fundamental principle behind FT-IRS lies in the interaction between infrared radiation and the vibrations of molecules. Each functional group within a molecule possesses a unique vibrational energy, and when infrared light of a matching frequency interacts with a molecule, it can cause changes in the molecule’s dipole moment, resulting in the absorption of the radiation (13). This absorption pattern, analyzed across a spectrum of infrared frequencies, generates a characteristic “fingerprint” that reflects a unique absorption profile. The combined spectral absorptions of molecules like lipids, proteins, and carbohydrates produce a distinctive fingerprint for each microbial cell (14–16), a highly specific IR signature applicable for identification from the genus to the species level and even down to serogroup/serotype and strain level (12, 14, 17–19). Given the discriminatory capability comparable to molecular genetic methods, it has also successfully been used for management of nosocomial outbreaks and in routine surveillance for bacterial relatedness (15, 18, 20–23).

This study investigated the application of the IRBT, a commercially available FT-IRS-based system for microbial typing, to differentiate *C. botulinum* strains based on their BoNT-encoding-gene type. Exploratory data analysis was conducted to examine various wave number regions, and machine learning models were developed and evaluated.

## MATERIALS AND METHODS

### Bacterial isolates

A total of 110 strains of botulinum neurotoxin-producing clostridia (BNPC) were analyzed. This included four reference strains (ATCC 19397, type A; ATCC 27765, type B; NCTC 10281, type F; and NCTC 8265, type D), 95 strains isolated from different animal species (wild birds, commercial poultry, and bovines) or animal feed (14 type C, 3 type D, 47 type C/D, and 31 type D/C), and 11 strains isolated from human outbreaks or food intended for human consumption (1 type A and 10 type B). The majority of the strains were isolated in the north of Italy between 2011 and 2024. Forty-three of the 95 animal strains originated from 14 outbreaks, with samples collected from animals, feed, and environment. Table 1 provides detailed information on all tested strains. BoNT types were determined using the multiplex real-time PCR method developed by the National Reference Center for Botulism, as previously described (2). The study also included a reference strain of *Clostridium novyi* (ATCC 25758).

**TABLE 1:** List of strains analyzed in the study^*a*^^,^^*b*^

Strain ID	BoNT type	Collection year	Animal species	Matrix of isolation	Outbreak
NCTC 8265	D	/	*C. botulinum – Ref. strain*	*/*	*–*
ATCC 19397	A	/	*C. botulinum – Ref. strain*	*/*	*–*
ATCC 27765	B	/	*C. botulinum – Ref. strain*	*/*	*–*
NCTC 10281	F	/	*C. botulinum – Ref. strain*	*/*	*–*
ATCC 25758	Non toxigenic	/	*C. novyi – Ref. strain*	*/*	*–*
10380/13/14	D/C	2014	*Bos taurus*	Feces	1
10380/17/14	D/C	2014	*Bos taurus*	Intestinal content (rectum)	
10380/18/14	D/C	2014	*Bos taurus*	Intestinal content (colon)	
10380/20/14	D/C	2014	*/*	Feed	
2454/7/14	D/C	2014	*Bos taurus*	Feces	2
2667/2/14	D/C	2014	*/*	Drinking water	
2454/6/14	D/C	2014	*Bos taurus*	Feces	
3522/5/13	D/C	2013	*Bos taurus*	Feces	3
3522/4/13	D/C	2013	*Bos taurus*	Feces	
3524/3/13	D/C	2013	*Bos taurus*	Intestinal content	
4888/20	D/C	2020	*Coturnix coturnix*	Intestinal content	4
5291/1/20	D/C	2020	*/*	Drinking water	
6126/2/20	D/C	2020	*/*	Drinking water	5
6126/11/20	D/C	2020	*Coturnix coturnix*	Intestinal content	
6126/12/20	D/C	2020	*Coturnix coturnix*	Intestinal content	
6126/20	D/C	2020	*Coturnix coturnix*	Intestinal content	
10140/23	D/C	2023	*Bos taurus*	Feces	*–*
511/6/22	D/C	2022	*Bos taurus*	Feces	*–*
6025/15	D/C	2015	*Ciconia ciconia*	Intestinal content	*–*
3859/5/11	D/C	2011	*Bos taurus*	Feces	*–*
7574/5/2012	D/C	2012	*Bos taurus*	Feces	*–*
4045/5/13	D/C	2013	*Bos taurus*	Liver	*–*
5257/23	D/C	2023	*Bos taurus*	Intestinal content	*–*
4456/11/10	D/C	2010	*Bos taurus*	Feces	*–*
1585/18/11	D/C	2011	*Bos taurus*	Rumen	6
1585/19/11	D/C	2012	*Bos taurus*	Rumen	
3859/5/11	D/C	2011	*Bos taurus*	Feces	*–*
5274/2/21	D/C	2021	*Bos taurus*	Feces	*–*
184/4/20	D/C	2020	*Bos taurus*	Rumen	*–*
5262/6B/14	D/C	2014	*Bos taurus*	Feces	*–*
1671/2/11	D/C	2011	*Bos taurus*	Ruminal content	*–*
6759/23	D	2023	*Alectoris rufa*	Intestinal content	*–*
6732/23	D	2023	*Perdix perdix*	Intestinal content	*–*
4150/24/18	D	2018	/	Feed	*–*
6433/2/13	C	2013	*Bos taurus*	Feces	7
6433/3/13	C	2013	*Bos taurus*	Feces	
6433/4/13	C	2013	*Bos taurus*	Feces	
6433/6/13	C	2013	/	Feed	
9877/1/12	C	2012	*Bos taurus*	Feces	8
9877/3/12	C	2012	*Bos taurus*	Feces	
10018/12	C	2012	*Bos taurus*	Feed	
10019/12	C	2012	*Felis silvestris*	Carcass	
10020/12	C	2012	*Bos taurus*	Feces	
5174/17/17	C	2017	*Cygnus cygnus*	Intestinal content	*–*
900/3/11	C	2011	*Bos taurus*	Liver	*–*
1792/1/18	C	2018	*Bos taurus*	Feces	*–*
9952/2/18	C	2018	*Bos taurus*	Feces	*–*
7853/23	C	2023	*Gallus gallus*	Intestinal content	*–*
2659/1/10	C/D	2010	*Gallus gallus*	Intestinal content	9
2659/2/10	C/D	2010	*Gallus gallus*	Intestinal content	
4879/20/10	C/D	2010	*/*	Rice hulls	
4879/10	C/D	2010	*/*	Rice hulls	
6503/1/13	C/D	2013	*Phasianus colchicus*	Liver	10
7494/7/14	C/D	2014	/	Soil of pheasant farm	
5993/15	C/D	2015	*Phasianus colchicus*	Intestinal content	11
5993/15	C/D	2015	*Phasianus colchicus*	Liver	
7465/19	C/D	2019	*Anas platyrhynchos*	Intestinal content	12
7468/19	C/D	2019	*Anas platyrhynchos*	Intestinal content	
10307/51/20	C/D	2020	*Gallus gallus*	Intestinal content	13
10307/48/20	C/D	2020	*Gallus gallus*	Liver	
4756/2/22	C/D	2022	*Gallus gallus*	Intestinal content	*–*
4944/22	C/D	2022	*Anas platyrhynchos domesticus*	Liver	*–*
10862/1/23	C/D	2023	*Gallus gallus*	Liver	*–*
7376/23	C/D	2023	*Gallus gallus*	Intestinal content	*–*
3636/2/22	C/D	2022	*Gallus gallus*	Intestinal content	*–*
4916/14/22	C/D	2022	*Anas platyrhynchos domesticus*	Intestinal content	*–*
2738/3/22	C/D	2022	*Gallus gallus*	Intestinal content	*–*
8439/2023	C/D	2023	*Gallus gallus*	Intestinal content	*–*
7338/2023	C/D	2023	*Gallus gallus*	Intestinal content	*–*
4998/23	C/D	2023	*Gallus gallus*	Liver	*–*
6111/23	C/D	2023	*Anas platyrhynchos domesticus*	Organ pool	*–*
6114/23	C/D	2023	*Fulica atra*	Organ pool	*–*
6116/23	C/D	2023	*Anas platyrhynchos*	Organ pool	*–*
6678/23	C/D	2023	*Gallus gallus*	Liver	*–*
6222/23	C/D	2023	*Gallus gallus*	Organ pool	*–*
8103/09	C/D	2009	*Gallus gallus*	Intestinal content	–
5792/11	C/D	2011	*/*	Rice hulls	*–*
3650/1/13	C/D	2013	*Gallus gallus*	Liver	*–*
7167/2012	C/D	2012	*Gallus gallus*	Intestinal content	*–*
5313/1/14	C/D	2014	*Gallus gallus*	Intestinal content	*–*
7608/2/14	C/D	2014	*Gallus gallus*	Intestinal content	*–*
6660/6/15	C/D	2015	*Gallus gallus*	Intestinal content	*–*
9659/15	C/D	2015	*Gallus gallus*	Intestinal content	*–*
6899/16	C/D	2016	*Gallus gallus*	Intestinal content	*–*
7343/3/18	C/D	2018	*Gallus gallus*	Liver	*–*
2974/2/20	C/D	2020	*Gallus gallus*	Intestinal content	*–*
3566/A/20	C/D	2020	*Gallus gallus*	Intestinal content	*–*
5792/1/15	C/D	2015	*Meleagris gallopavo*	Liver	14
5792/2/15	C/D	2015	*Meleagris gallopavo*	Intestinal content	
1580/24	C/D	2024	*Meleagris gallopavo*	Stomach content	*–*
1874/20/24	C/D	2024	*Gallus gallus*	Feed	*–*
5075/3/24	C/D	2024	*Anas platyrhynchos domesticus*	Intestinal content	*–*
5288/24	C/D	2024	*Gallus gallus*	Intestinal content	*–*
5641/2/24	C/D	2024	*Gallus gallus*	Stomach content	*–*
5925/1/24	C/D	2024	*Gallus gallus*	Liver	*–*
1/21	B	2021	/	Vegetable and grain soup	*–*
307/20	B	2020	/	Boiled greens	*–*
318/20	A	2020	/	Boiled greens	*–*
69/3/21	B	2021	/	Enema	*–*
10564/23	B	2023	Human	Rectal swab	*–*
283/2/21	B	2021	Human	Intestinal content	*–*
427/3/23	B	2023	*/*	Pepper sauce	*–*
5182/2/21 B	B	2021	Human	Feces	*–*
5628/5/23	B	2023	Human	Rectal swab	*–*
5628/7/23	B	2023	Human	Rectal swab	*–*
8319/20 B	B	2020	Human	Enema	*–*

^*a*^ A slash (“/”) in the “matrix of isolation” column means unknown matrix of isolation, the samples consist of reference strains; A slash (“/”) in the “animal species” column denotes non-animal isolation. Detailed isolation matrices are listed in the “matrix of isolation” column. A slash (“/”) in the “collection year” column means unknown year of isolation, the samples consist of reference strains.

^*b*^ “–” indicates non outbreak related strain.

### Sample preparation

Strains were cultured on Blood Agar Base (BAB) at 37°C ± 2°C for 48 ± 2 h in an anaerobic cabinet (Shel Lab, Cornelius, OR, USA) with an atmosphere composed of 5% hydrogen, 5% carbon dioxide, and 90% nitrogen. Sample preparation for IRBT (Bruker Daltonics GmbH and Co. KG) analysis was performed according to the manufacturer’s instructions. Briefly, a 1 µL loop of bacterial cultures was collected and suspended in 50 µL of 70% (vol/vol) ethanol solution in a suspension vial (Bruker Daltonics, Bremen, Germany). Following the addition of 50 µL of ultrapure water, samples were homogenized by vortexing. Subsequently, 15 µL of the bacterial suspension was spotted in four technical replicates onto the 96-spot silicon IRBT target and allowed to dry for 15–20 min at room temperature in a normal atmosphere. For each run, quality control was performed using the Infrared Test Standards IRTS 1 and 2 provided in the IRBT kit (Bruker Daltonics, Bremen, Germany). All steps for IRBT sample preparation and measurements were carried out on a standard laboratory bench, under ambient laboratory conditions without specific control of temperature or humidity.

### Spectra acquisition and data analysis

Spectra were acquired and processed using OPUS v8.2 and IRBT Client software v3.1 (Bruker Daltonics, Bremen, Germany) using default settings recommended by the manufacturer. For the first batch of isolates (*n* = 30), the spectra acquisition was performed in Istituto Zooprofilattico Sperimentale delle Venezie, while the second batch of isolates was measured in Bruker Daltonics’ laboratory in Bremen. Spectra were acquired in the whole mid-IR range (4,000–500 cm^−1^). After acquisition, the second derivative of the absorption values was calculated over nine data points using the Savitzky-Golay algorithm. The spectra were then vector-normalized and subsequently interpolated to have one data point for each integer wavenumber. The spectral regions corresponding to the IR radiation absorption of the main biological macromolecules (carbohydrates: 1,200–900 cm^−1^; lipids: 3,200–2,800 cm^−1^ and 1,500–1,400 cm^−1^; proteins: 1,800–1,500 cm^−1^) were investigated to find the most discriminatory ones. The spectral region from 1,800 to 700 cm^−1^ was found to provide the best discriminatory power, and therefore, it was used to perform similarity analysis by HCA, PCA, and LDA, which are algorithms implemented into the IRBT software for an automated application.

### Machine learning and development of automated classifiers

The artificial intelligence capabilities of the IRBT software were applied to develop a classifier for *C. botulinum* differentiation at the serogroup and subtype level. Three different machine learning algorithms were tested: linear SVM, RBF SVM, and ANN. These algorithms, well established, which come from openly accessible Java libraries, are implemented into the IRBT software. For each one of them, only a few parameters can be set by the users (cost factor [C] for both linear and RBF SVM, and number of training cycles for ANN). The machine learning algorithms were trained with a set of well-characterized isolates to “learn” to recognize the specific characteristics of each class (toxin types, in this case). A four-fold cross-validation was performed to verify the predictive accuracy. The classification process relies then on the attribution of one of toxin types included in the training data set to unknown samples, according to the model calculated by the algorithm. The classification result was delivered with a “traffic light” color code scoring system (green-yellow-red), which is related to the reliability of the classification. The threshold values applied to define the three categories were extrapolated from the distribution of the outlier values of the validation cohort of samples, considering the Youden index. A “green score” result means that the sample spectrum is located within the spectral space of the training set (high reliability). A “yellow score” result means that the sample spectrum is located at the periphery of the spectral space of the training set (moderately reliable). A “red score” value means that the sample spectrum is located far away from the spectra of any class included in the training set; therefore, it cannot be considered reliable.

In this study, a machine learning approach was applied to animal origin strains and one *C. botulinum* type D reference strain to build a classifier to distinguish the different toxin types in the group III of *C. botulinum*. A total of 32 isolates (*n* = 2 D, *n* = 9 C, *n* = 11 CD, and *n* = 10 DC), chosen in order to represent the variance within the respective group, were used as training set, representing each group and the variance within each group. The remaining 64 strains were used as a testing set (*n* = 2 D, *n* = 5 C, *n* = 36 CD, and *n* = 21 DC).

## RESULTS

### Exploratory data analysis

**Fig 1:**
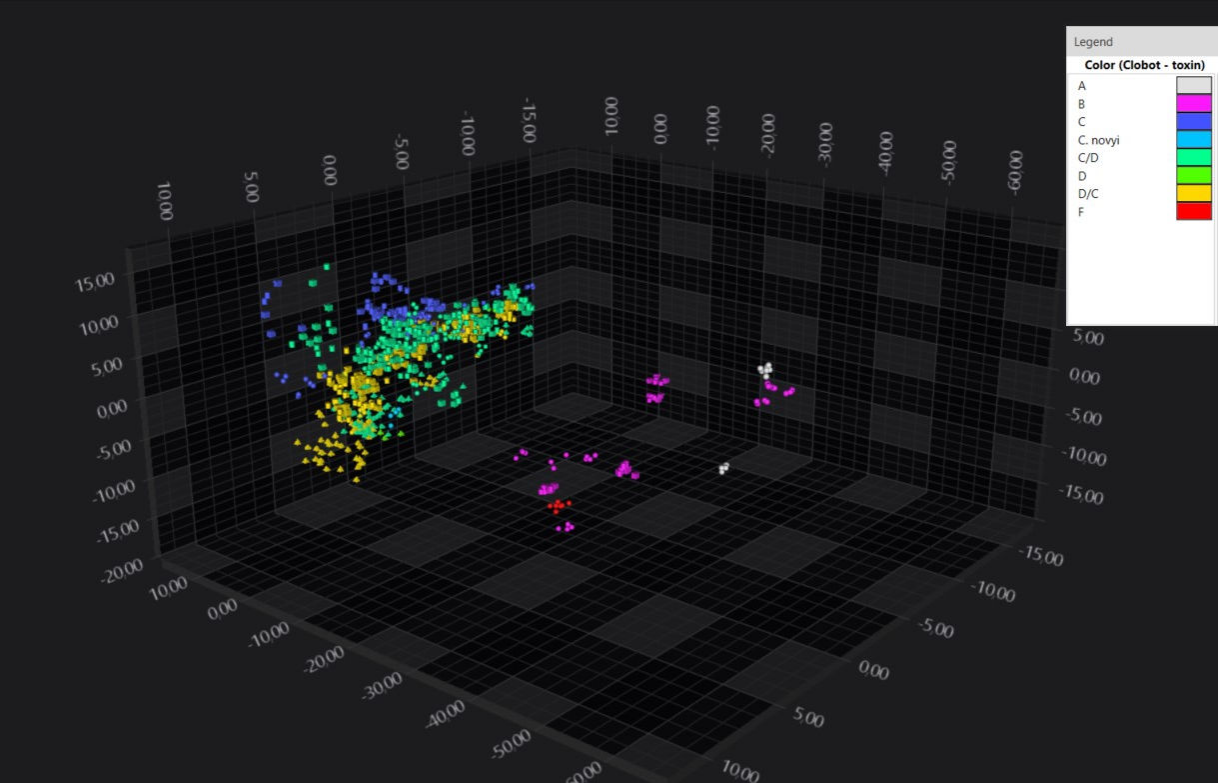
LDA 3D scatterplot illustrating the distinct separation of *C. botulinum* phylogenetic group III (yellow, blue, and green) and I (purple, red, and gray) within the spectral space. Each geometric form represents a spectrum (all replicates were plotted) with color indicating different BoNT types.

The examination of the full data set in the 1,200–900 cm⁻¹ spectral region corresponding to the carbohydrates absorption revealed the highest discriminatory power for distinguishing *C. botulinum* phylogenetic groups. Indeed, PCA/LDA scatterplot analysis yielded a well-defined separation between type A, B, and F strains belonging to *C. botulinum* phylogenetic group I and group III strains comprising type C, CD, DC, and D types (Fig. 1). Notably, group I strains displayed considerable heterogeneity, and *C. novyi* clustered with group III strains.

**Fig 2:**
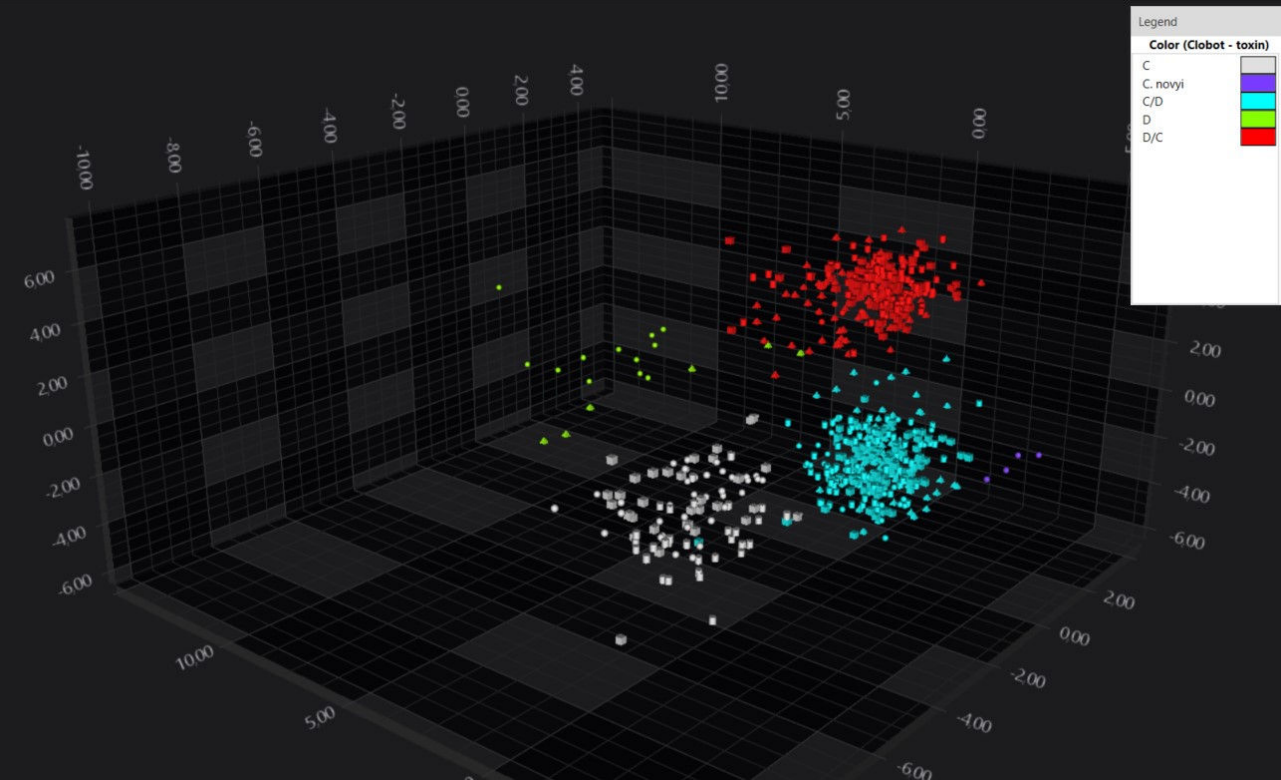
LDA 3D scatterplot, which shows the clear separation in the multidimensional spectral space of the four toxin types comprised *C. botulinum* group III. Geometric shapes represent individual spectra (all replicates were plotted) with color indicating different BoNT types.

For *C. botulinum* strains belonging to type C, D, CD, and DC, the 1,800–1,500 cm^−1^ spectral region associated with amide group absorption demonstrated the highest discriminatory power for distinguishing among these toxin types. Indeed, types C, CD, and DC strains formed three distinct clusters, and type D isolates displayed significant diversity but remained within a shared spectral area (Fig. 2).

**Fig 3:**
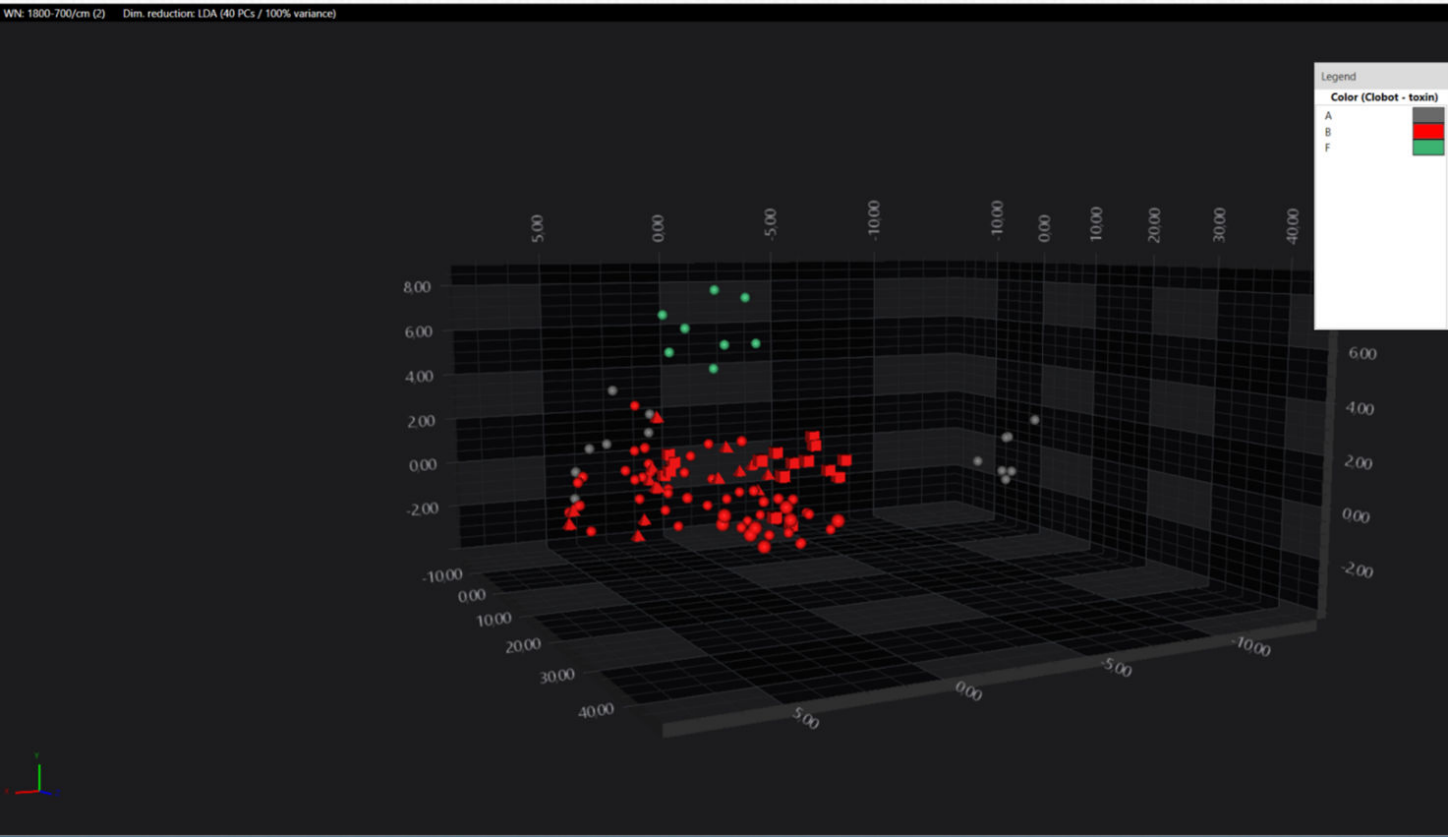
3D LDA scatterplot showing the separation in the spectral space of the toxin type F from toxin types A and B. Geometric shapes represent individual spectra (all replicates were plotted) with color indicating different BoNT types.

Clear discrimination between *C. botulinum* toxin type F and types A and B was observed upon analysis of strains A, B, and F within the 1,800–700 cm⁻¹ spectral region, associated with amide groups, carbohydrates, and fatty acid absorption. Notably, one type A isolate was clearly distinguishable from the others (Fig. 3).

### Machine learning

**Fig 4:**
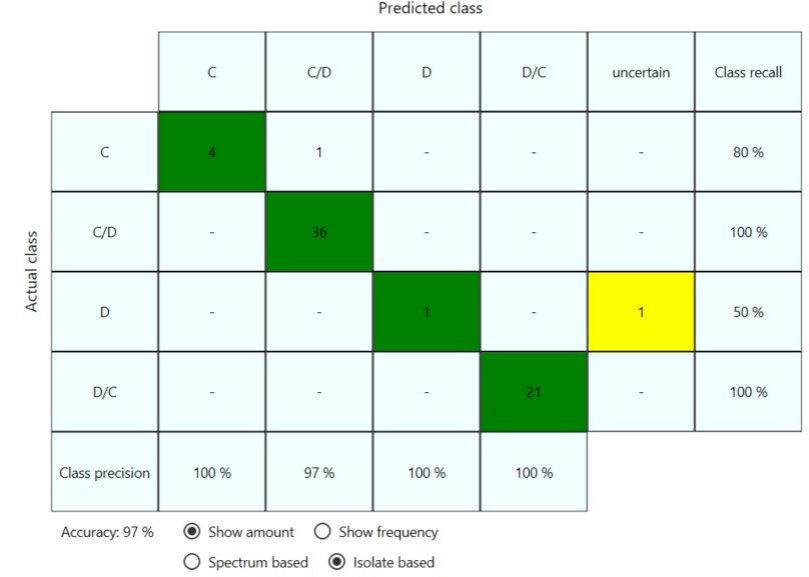
Performance of the SVM classifiers on the testing set. The numbers in the box correspond to the number of isolates.

For the classifiers developed using SVM algorithms (linear and RBF), the C value was 50 and 10, respectively. For the classifier developed using ANN, 400 training cycles were performed. Among the three machine learning algorithms tested, the classifier created with the linear SVM algorithm showed the highest performance, achieving 97% accuracy (62/64 isolates) and 3% of error rate on the testing set. Specifically, only one type C isolate was misclassified as CD, and the type D reference strain NCTC 8265 was classified as uncertain. The ANN and RBF SVM algorithms exhibited lower accuracies, 74% and 92.3%, respectively. Fig. 4 presents the confusion matrix, illustrating the performance of the SVM classifier on the testing set. The classifier’s specificity, as represented by the class precision values, was 100% for type C, D, and DC strains and 97% for CD strains. Sensitivity was 100% for *C. botulinum* type DC and CD, 80% for type C, and 50% for type D.

**Fig 5:**
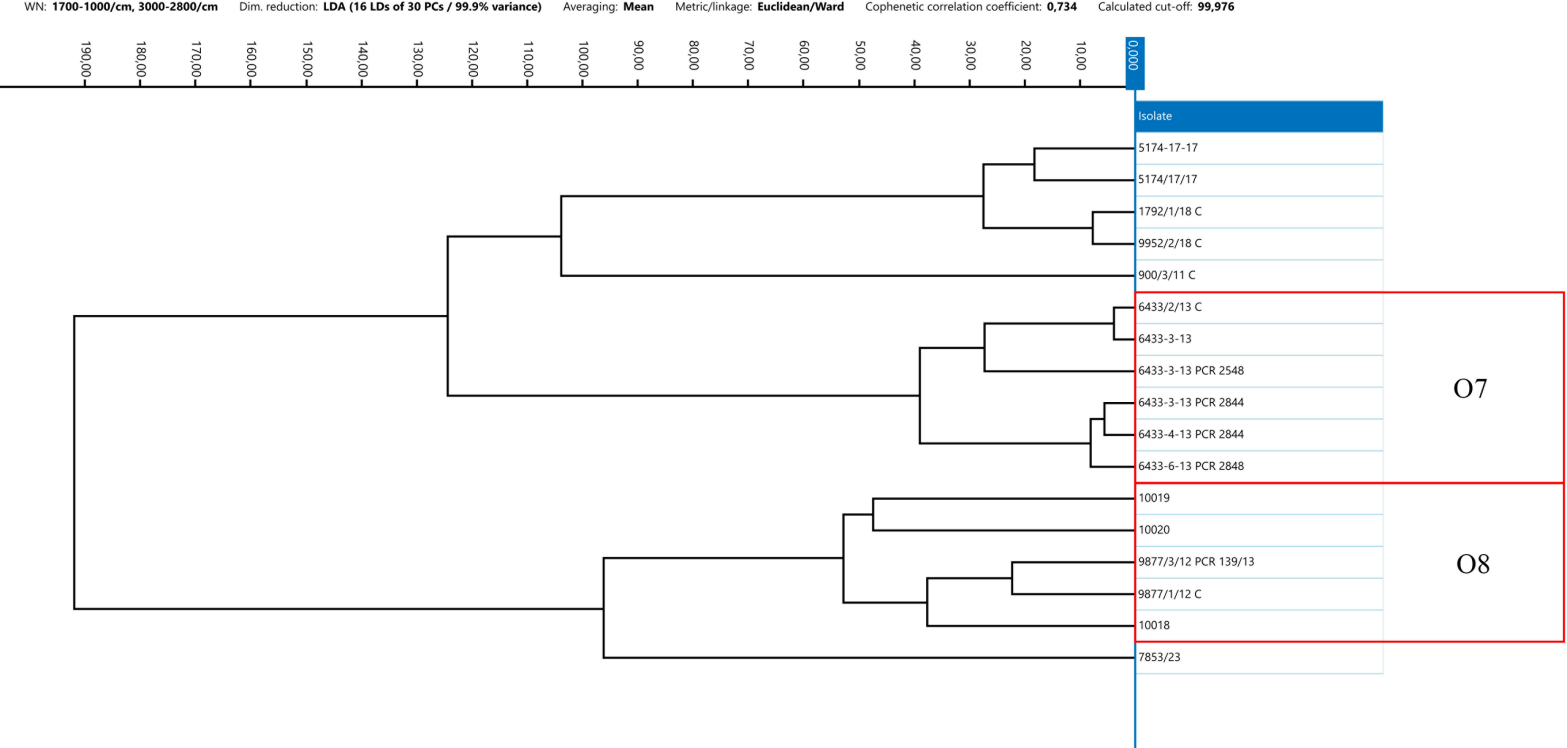
Dendrogram resulted from the clustering analysis of spectra obtained from type C strains. Spectra were pre-processed by LDA applied at the isolate level, using 30 Principal Components, Euclidean metric, and Ward’s algorithm as linkage type. Every isolate is represented by its average spectrum, automatically calculated. O = outbreak.

**Fig 6:**
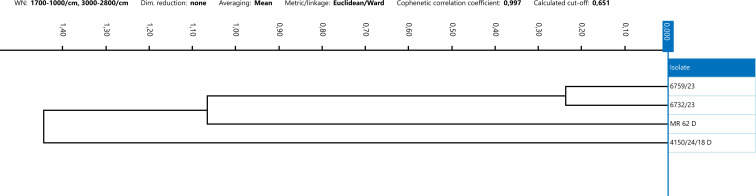
Dendrogram resulted from the clustering analysis of spectra obtained from type D strains. Spectra were analyzed applying Euclidean metric and Ward’s algorithm as linkage type. Every isolate is represented by its average spectrum, automatically calculated.

**Fig 7:**
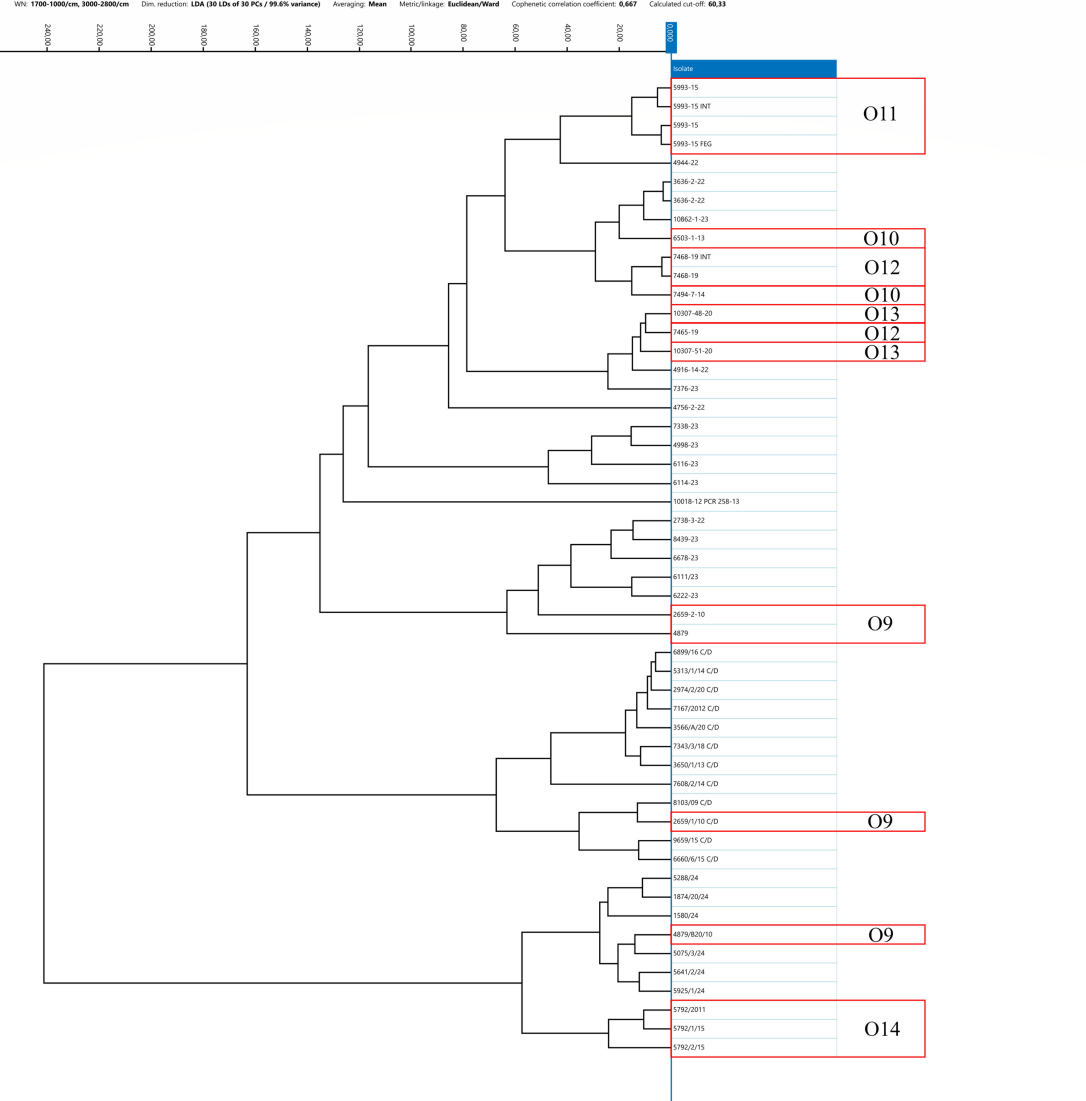
Dendrogram resulted from the clustering analysis of spectra obtained from type CD strains. Spectra were pre-processed by LDA applied at the isolate level, using 30 Principal Components, Euclidean metric, and Ward’s algorithm as linkage type. Every isolate is represented by its average spectrum, automatically calculated.

**Fig 8:**
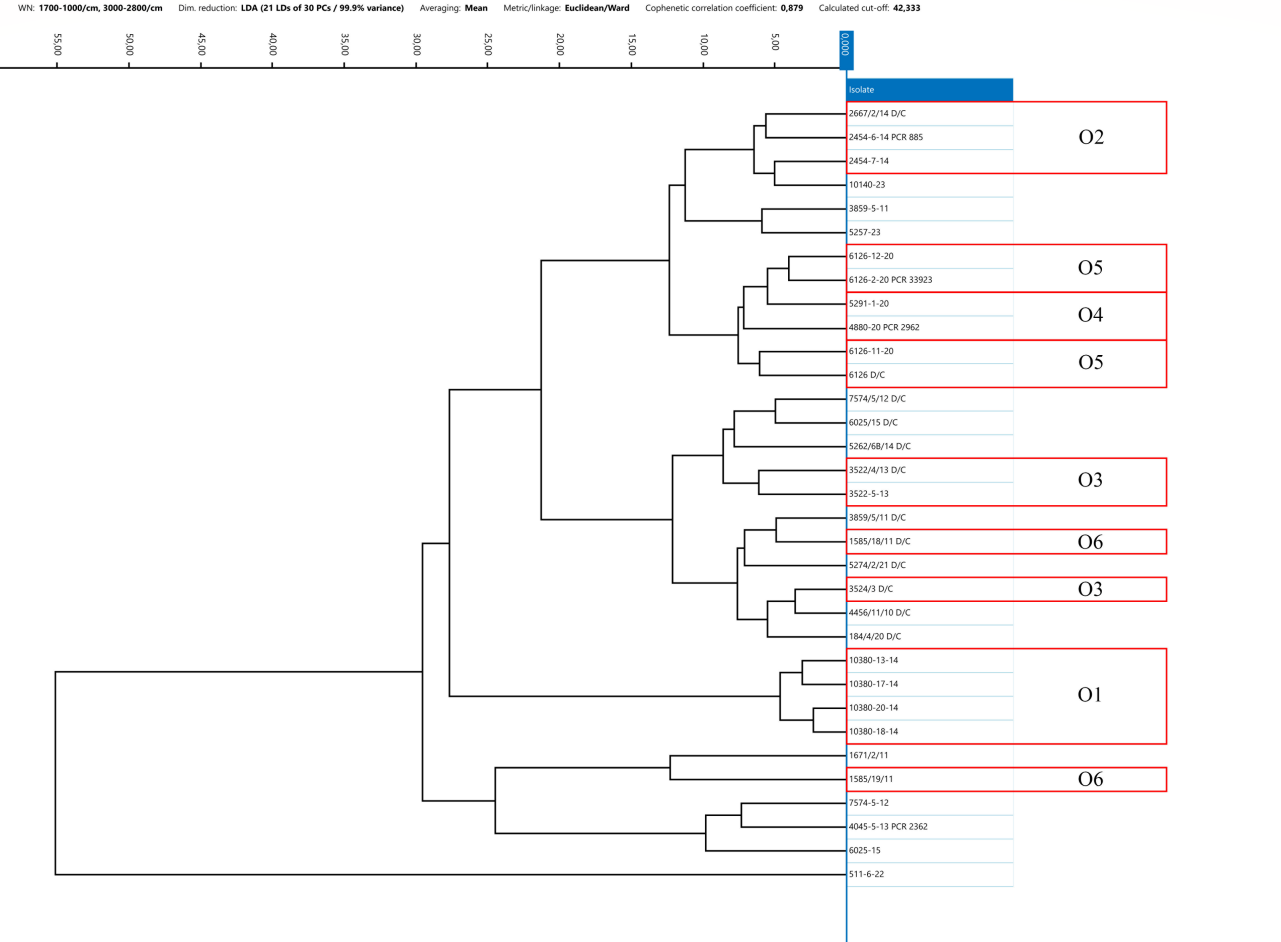
Dendrogram resulted from the clustering analysis of spectra obtained from type DC strains. Spectra were pre-processed by LDA applied at the isolate level, using 30 Principal Components, Euclidean metric, and Ward’s algorithm as linkage type. Every isolate is represented by its average spectrum, automatically calculated. O = outbreak.

The IRBT system’s strain differentiation capability within a single outbreak was evaluated by analyzing *C. botulinum* isolates of types C, CD, and DC collected during the same animal outbreak from samples collected from both animals and the suspected source of contamination (feed and environment, e.g., water and soil). Several outbreak strains were previously analyzed using a WGS approach. For 10 out of 14 groups of strains isolated from the same outbreak, specifically strains belonging to outbreaks 1, 2, 4, 5, 7, 8, 10, 11, 13, and 14, FT-IRS demonstrated strong similarity within the spectra (24). Notably, *C. botulinum* from outbreaks 4 and 5 exhibited highly similar IR spectra. For outbreaks 1, 2, 7, 8, 10, and 14, the epidemiological link between the strains was confirmed also by WGS results, whereas WGS data were not available for the remaining outbreaks 4, 5, 11, and 13. In contrast, for 4 to 14 epidemiologically linked *C. botulinum*, specifically strains belonging to outbreaks 3, 6, 9, and 12, FT-IRS did not reveal a clear similarity among the IR spectra (Fig. 5 to 8). Furthermore, sequencing data of clusters 6 and 9 revealed genetic divergences. In outbreak 3, WGS and FT-IRS results were discordant. No WGS data were available for outbreak 12.

## DISCUSSION

FT-IRS has proved to be a powerful and versatile analytical technique within the field of microbiology (12). For complex biological samples such as microorganisms, the spectral fingerprint resulted from the FT-IRS analysis reflects the overall biochemical composition of the cell, encompassing proteins, lipids, carbohydrates, and nucleic acids (25). FT-IRS has been widely studied for strain typing and subtyping, which is crucial for epidemiological investigations and outbreak management (14). FT-IRS method displayed better discriminatory power than Pulsed-Field Gel Electrophoresis (PFGE) for sub-typing *E. coli* O157:H7 (26) showed to be very promising and user-friendly tool for *Salmonella* typing at serogroup level (22) and for discrimination of *Listeria monocytogenes* at serotype, haplotype, and strain levels (27). Several studies have demonstrated the congruence of FT-IRS-based typing with gold-standard methods in outbreak scenarios. By analyzing subtle differences in the spectral fingerprints of bacterial isolates, it can effectively cluster related strains and identify potential sources of infection during outbreaks (28). FT-IRS demonstrated the greatest utility as a first screening tool for evaluating epidemiological correlation between different isolates of *Enterobacterales*, *Staphylococcus aureus*, *Legionella pneumophila*, and *Acinetobacter baumannii* in outbreak investigations (29–33). It has been also successfully used to differentiate between antibiotic-resistant and susceptible strains of various bacterial species. The technique can also monitor metabolic changes occurring in bacteria in response to antibiotic exposure, providing insights into the mechanisms of resistance. Furthermore, the integration of machine learning algorithms has significantly enhanced the ability to rapidly and accurately predict antibiotic susceptibility (21, 34, 35).

Laboratory diagnosis of botulism relies on a combination of clinical assessment and laboratory testing to confirm the presence of either BoNT or BNPC. Primary laboratory methods focus on detecting the toxin directly in clinical or food samples using techniques such as the mouse bioassay, immunological assays (e.g., ELISA), and the recently developed EndoPep-MS. Complementing these, molecular techniques such as conventional or real-time PCR are used to detect BNPC by amplifying BoNT genes, often directly from enrichment broth cultures (36, 37). A key advantage of molecular methods is the ability to determine not only the BoNT serotype but also potentially identify subtype or mosaic toxin forms, often without the need for prior isolation of bacterial strains. These specialized diagnostic methods are typically performed only in a few reference laboratories; therefore, it would be valuable to have methods that are also accessible to routine clinical diagnostic laboratories. Once botulism has been confirmed, it is essential to determine the source of contamination to prevent further illness. Thus, over the years, a number of molecular typing tools have been developed for the genetic characterization and epidemiological investigation during botulism outbreaks, especially for group I and II strains (36). These include PFGE, and ribotyping, as well as PCR-based techniques such as Amplified Fragment Length Polymorphism (AFLP), Randomly Amplified Polymorphic DNA analysis (RAPD), Repetitive Element sequence-based PCR (Rep-PCR), Multilocus Sequence Typing (MLST), Multi-locus Variable Number Tandem Repeat Analysis (MLVA), and DNA sequencing (36, 37). Although PFGE analysis has been widely used for investigating foodborne outbreaks, it requires prior strain isolation, is labor-intensive and time-consuming, and necessitates specialized equipment (38). Moreover, not all *C. botulinum* strains, particularly those belonging to Group II, are typeable using standard PFGE protocols due to the presence of endogenous DNases. AFLP and RAPD exhibit discriminatory power comparable to that of PFGE, but their low reproducibility makes standardization both challenging and resource-intensive. Finally, MLVA offers a significant advantage over the above-mentioned methods, as it does not require strain isolation and can be performed directly on DNA extracted from the initial sample. It is particularly well suited for addressing micro-evolutionary questions in outbreak investigations (39). However, the method is relatively expensive and requires access to molecular biology infrastructure. Our group also investigated the use of matrix-assisted laser desorption/ionization time-of-flight mass spectrometry (MALDI-TOF MS) as a potential tool for *C. botulinum* Group III outbreak investigations, but it proved inadequate for this purpose (10). While some of the aforementioned methods are still in use, there is a growing trend toward adopting sequencing-based approaches for outbreak investigations. Sequencing offers the highest resolution and discriminatory power for accurate source tracking. Nevertheless, the entire workflow, from sample collection and bacterial isolation to DNA extraction, sequencing, and complex bioinformatics analysis, can still require several days to weeks to complete, even with faster technologies. Moreover, the substantial cost of sequencing reagents, along with the required investment in specialized equipment, high-performance computing infrastructure, data storage, and dedicated bioinformatics expertise for data analysis and interpretation continues to limit this technique’s accessibility to only a select number of laboratories. FT-IRS could offer several advantages for epidemiological investigations, including rapid turnaround time, low operational costs, non-destructive sample analysis, and the ability to perform strain-level discrimination without the need for complex molecular biology procedures or sequencing. To the best authors’ knowledge, this technology has not been previously evaluated for the characterization of *C. botulinum* at the toxin-serotype level. The only study in this area is by Kirkwood and colleagues (40), published in 2006. Their research demonstrated the capability of Focal Plane Array FT-IRS to distinguish between Group I and Group II BNPC.

In this study, we evaluated the possible use of the IRBT, a commercially available FT-IRS-based system, to distinguish *C. botulinum* strains based on their BoNT-encoding gene types and assess its applicability for epidemiological investigations.

PCA/LDA scatterplot analysis, consistent with lineages identified in WGS studies, revealed a clear separation between type A, B, and F strains (belonging to *C. botulinum* phylogenetic groups I/II) and group III strains comprising types C, CD, DC, and D with good clustering within each type. While good discrimination was observed between *C. botulinum* toxin type F, A, and B, the limited number of the analyzed strains suggests that a more comprehensive collection is needed for further investigation. Regarding type C, CD, DC, and D strains, they clustered into four well-defined groups, although type D isolates showed significant diversity among themselves. This observed diversity in type D strains can likely be attributed to the small number of analyzed strains (*n* = 4), with one of them being the non-toxigenic reference strain NCTC 8265 (also labeled MR 52 D in Fig. 6).

The SVM algorithm-based classifier showed good performance on identifying the BoNT type (97% accuracy), with sensitivity reaching ≥80% for *C. botulinum* types DC, CD, and C. Nevertheless, the sensitivity for type D was lower, probably as a result of the limited number of type D strains included, also in this case. Increasing the representation of type D strains would likely enhance the classifier’s training and performance for this type. However, type D strains remain extremely rare, as evidenced by the small number of published genomes available and the unavailability of a toxigenic reference strain (24). Despite this limitation, the study still offers valuable insights because it focused on types C, C/D, and D/C, which are the main causative agents of animal botulism.

The potential of FT-IRS as a rapid and inexpensive tool for epidemiological investigation of animal outbreaks was evaluated using C, CD, and DC strains from 14 outbreaks. FT-IRS analysis revealed a high degree of similarity in the IR spectra of case-related strains for most outbreaks (10/14). WGS data were available for 6 out to 10 of those outbreaks (24), and the genetic data confirmed the epidemiological link between strains. Results from isolates belonging to outbreaks 4 and 5 further supported the capacity of FT-IRS to detect spectral similarity in epidemiologically linked strains. These outbreaks, which occurred months apart at the same quail farm, thus confirmed a plausible epidemiological link. For four of the 14 outbreaks, FT-IRS did not reveal a clear epidemiological correlation among strains belonging to the same outbreak. In two of these four cases (outbreaks 6 and 9), this lack of correlation was also confirmed by WGS analysis. In another case (outbreak 3), the results from the two methods were discordant: FT-IRS identified a clear epidemiological relationship between only two of the three strains, whereas WGS analysis revealed a strong genetic correlation among all three strains. Overall, FT-IRS and WGS data were available for nine outbreaks, and the results were concordant in eight out of nine cases. Regarding outbreak 12, for which no genetic information is available, the strains were isolated from two wild mallard carcasses recovered in a hunting reserve. It is therefore plausible that the sources of contamination may differ, but further investigation through sequencing is needed to draw definitive conclusions.

In summary, FT-IRS demonstrated its ability to effectively distinguish between *C. botulinum* groups I/II and III, and within group III, to differentiate between mosaic and non-mosaic toxins. Moreover, although these results are still preliminary and require confirmation through further analyses involving a larger number of strains from the same and different outbreaks, the IRBT system has already proven to be a promising, user-friendly, and cost-effective tool for the epidemiological investigation of botulism outbreaks. The integration of machine learning algorithms could further enhance this approach, offering a novel and accessible method for BNPC typing that could be implemented even in laboratories without specific expertise in botulism investigation.

## Data Availability

All original data generated during the study are included in the article in the form of figures and tables. For further inquiries, please contact the corresponding author.
